# Assessment of intra- and inter-ventricular cardiac dyssynchrony in patients with repaired Tetralogy of Fallot: a cardiac magnetic resonance study

**DOI:** 10.1186/1532-429X-16-S1-P120

**Published:** 2014-01-16

**Authors:** Linyuan Jing, Christopher M Haggerty, Jonathan D Suever, Ashwin Prakash, Frank Cecchin, Oskar Skrinjar, Tal Geva, Andrew J Powell, Brandon K Fornwalt

**Affiliations:** 1 Department of Pediatrics, Physiology, Biomedical Engineering and Medicine, University of Kentucky, Lexington, Kentucky, USA; 2 Department of Cardiology, Boston Children's Hospital, Boston, Massachusetts, USA; 3 Department of Pediatrics, Harvard Medical School, Boston, Massachusetts, USA; 4 Scientific Imaging and Visualization LLC, Atlanta, Georgia, USA

## Background

Patients with repaired tetralogy of Fallot (TOF) frequently have right bundle branch block. However, the contribution of cardiac dyssynchrony to dysfunction remains controversial. To better understand this phenomenon and ultimately study therapies, we developed a method to quantify left (LV), right (RV) and inter-ventricular cardiac dyssynchrony using standard cine CMR.

## Methods

30 patients with repaired TOF (age 28 ± 16, 46% female) and 17 healthy controls (age 29 ± 7, 12% female) underwent cine CMR. Patients were imaged twice to assess inter-test reproducibility. Circumferential strain vs time curves were generated with a custom feature tracking algorithm for 12 LV and 12 RV segments in 4-7 slices encompassing the ventricles. For each segment, the temporal offset (TO) of the strain curve relative to a global reference curve derived from the controls was calculated and expressed as a percent of the cardiac cycle. The intra-ventricular dyssynchrony index (DI) for each ventricle was computed as the standard deviation (SD) of the TOs (more dyssynchrony increases the SD). The inter-ventricular DI was calculated as the difference in median RV and median LV TOs. Regional dyssynchrony was quantified in 3 LV (septum, infero-lateral and antero-lateral wall) and 3 RV (septum, sinus, outflow tract) regions using median TOs.

## Results

**Figure 1:**
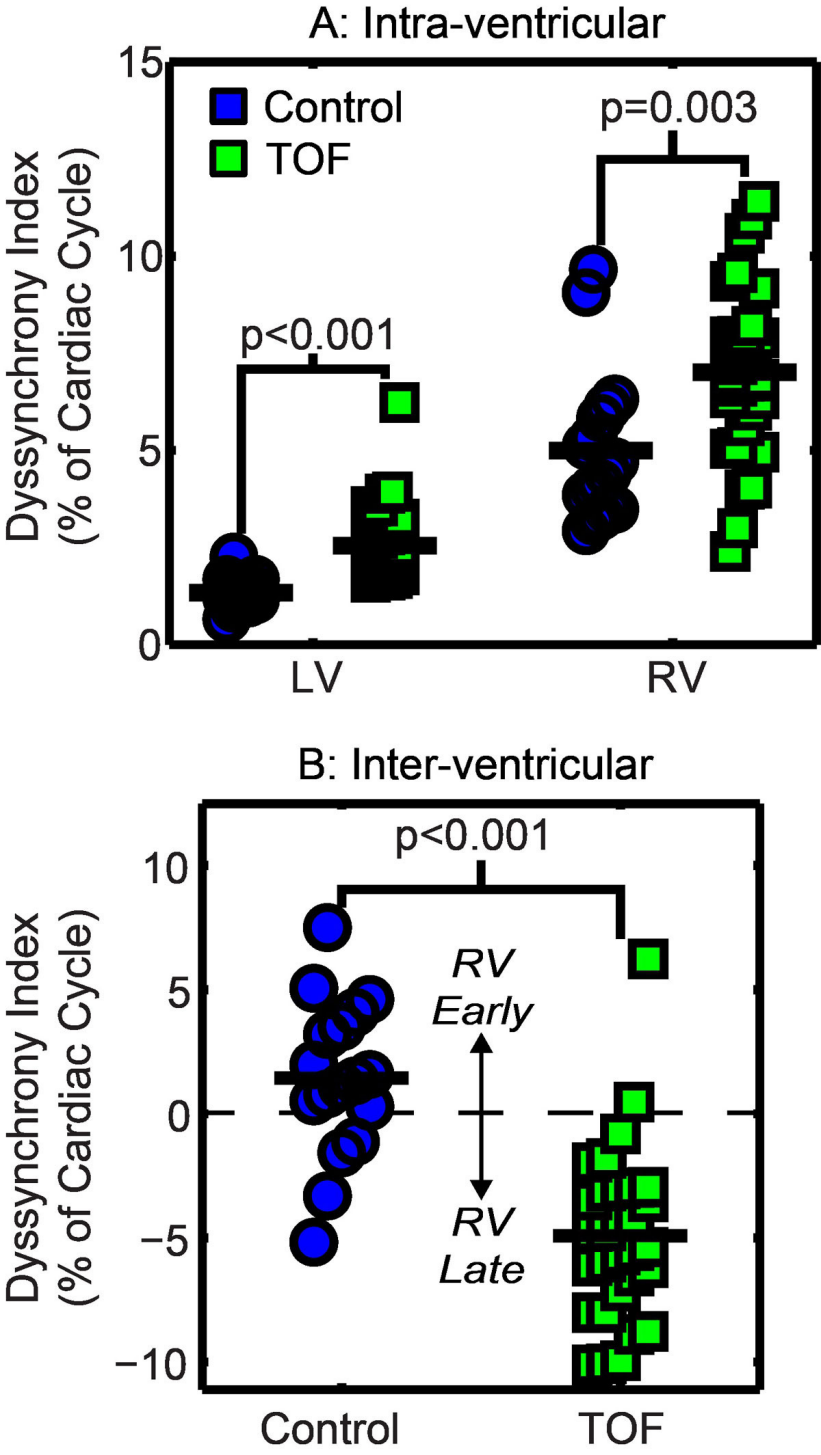
**Results for the three dyssynchrony indices (DIs) in controls (n = 17) and patients with repaired tetralogy of Fallot (TOF, n = 30)**. Note that for the inter-ventricular DI (B), positive values represent early RV contraction while negative values represent late RV contraction.

**Figure 2:**
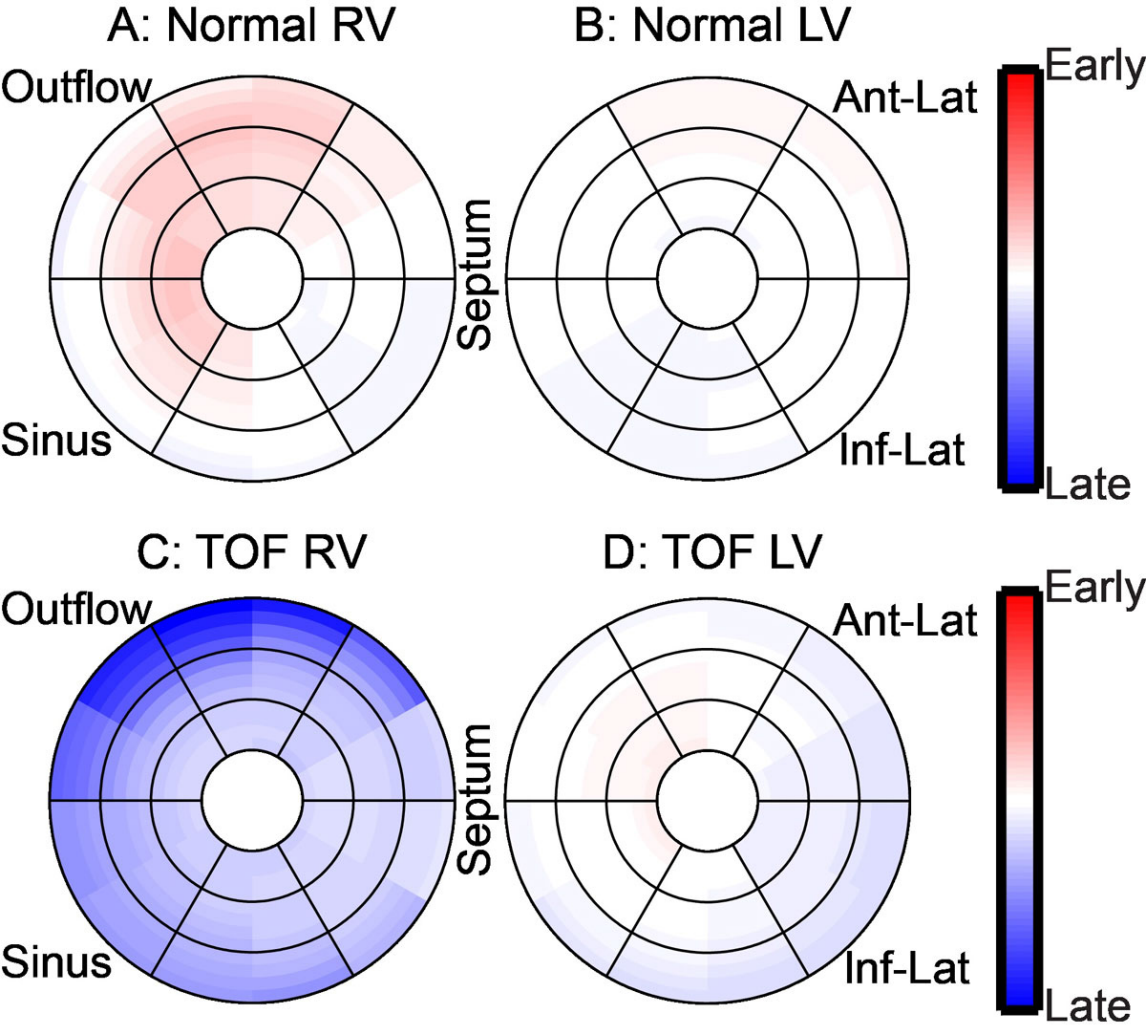
**Patients with repaired tetralogy of Fallot (TOF, n = 30) have a significantly different timing of contraction compared to controls (n = 17)**. Average temporal offsets are color coded from red (25% of the cardiac cycle earlier than the normal reference curve) to blue (25% later than the normal reference curve) and displayed on the bullseye.

Compared to controls, patients with repaired TOF had a greater LV, RV and inter-ventricular DI (Figure 1). The greater inter-ventricular delay in the patients was primarily due to a global delay in RV contraction with the RV contracting 4.9 ± 3.5% later than the LV in patients vs 1.4 ± 3.2% earlier in controls (Figure 1). Median TOs were similar in the three LV regions between patients and controls, but all three RV regions were significantly delayed in patients compared to the controls. Contraction patterns in the RV were also distinct: in controls, the earliest contraction was seen in the outflow tract; in patients, contraction occurred first in the septum and last in the outflow tract (Figure 2). Inter-test reproducibility for the three DIs was good with all coefficients of variation <20%. QRS duration was prolonged in the patients compared to the controls (150 ± 27 ms vs 85 ± 8 ms, p < 0.001). However, QRS duration was not correlated with any of the DIs.

## Conclusions

Patients with repaired TOF suffer from left, right and inter-ventricular cardiac dyssynchrony which can all be quantified from standard cine CMR with good inter-test reproducibility. Future studies need to determine whether these patients may benefit from resynchronization therapy.

## Funding

This work was supported by a National Institutes of Health (NIH) Director's Early Independence Award (1DP5OD012132-01), and NIH grant number KL2 RR033171 from the National Center for Research Resources and the National Center for Advancing Translational Sciences. The content is solely the responsibility of the authors and does not necessarily represent the official views of NIH.

